# Ointment for Itching Skin Diseases

**Published:** 1898-09

**Authors:** 


Ointment for Itching Skin Diseases. ℞.—Zinci oxidi, ʒiv; carbolic acid, grs. xij; salicylic acid, grs. xx; lanolin, ʒvj; vaselin, ℥ij. M.

